# Impact of Composting Methods on Nitrogen Retention and Losses during Dairy Manure Composting

**DOI:** 10.3390/ijerph16183324

**Published:** 2019-09-09

**Authors:** Xiao Yang, Enke Liu, Xinmeng Zhu, Hongyuan Wang, Hongbin Liu, Xiu Liu, Wenyi Dong

**Affiliations:** 1 Institute of Environment and Sustainable Development in Agriculture, Chinese Academy of Agricultural Sciences, Beijing 100081, China; 2 Institute of Agricultural Resources and Regional Planning, Chinese Academy of Agricultural Sciences, Beijing 100081, China

**Keywords:** dairy manure, composting methods, nitrogen losses, nitrogen emissions

## Abstract

Currently, composting is one of the most effective methods for treating fecal waste on large-scale livestock and poultry farms, but the quality effects of different composting methods are different. In this study, we implemented four composting methods, including farmer compost (FC), anaerobic compost (AnC), mixed compost (MC), and aerobic compost (AC), to study the effects of different composting methods on nitrogen (N) losses while composting dairy manure. Our results showed that the germination indexes (GIs) of three of the composting treatments (AnC, MC, and AC) exceeded 80%, which met the maturity requirements for composted products. Ammonia (NH_3_) emissions were the main contributor to nitrogen losses, while accumulated nitrous oxide (N_2_O) emissions accounted for the lowest proportion of nitrogen losses. The cumulative N losses via the leachate of the AC treatment were the lowest and accounted for 0.38% of the initial total nitrogen (TN). The accumulated N losses of the AC, FC, AnC, and MC treatments accounted for 13.13% 15.98%, 15.08%, and 19.75%, respectively, of the initial TN. Overall, the AC method significantly reduced N losses via leachates, further reducing TN losses. This observation suggests that AC might be an appropriate method for highly efficient nitrogen management during dairy manure composting.

## 1. Introduction

According to data released by the United States Department of Agriculture (USDA), there were 144 million cows in the world in 2018. Furthermore, the amount of dairy manure generated in China has increased dramatically with the rapid development of the dairy farming industry. Overproduction has generally led to inappropriate manure disposal practices [1,2]. Although fresh dairy manure is a valuable resource for organic fertilizer because of its high proportion of biodegradable organic compounds and nutrient (N and P) contents, it is unsuitable for direct land application because fresh manure contains pathogens and weed seeds and is difficult to preserve and transport [3,4].

As a sustainable approach for addressing fecal waste, composting is currently encouraged prior to applying manure to land, and composting can reduce the volume and mass of manure as well as provide safer and more stable organic fertilizer or soil amendments [1,5,6]. Compost is used as a supply of nitrogen [7]. However, nitrogen losses are also associated with composting, such as those through gases (mainly NH_3_ and N_2_O) and leachates, which cause secondary environmental pollution [8,9,10]. NH_3_ emissions are also notorious for their odor and toxicity [11,12], and N_2_O is a major greenhouse gas. The leachate discharged during composting is regarded as a pollution source in soil, surface water, and groundwater when considering its substantial potential pollutants, such as organic substrates and pathogens. Moreover, these gases and leachates are also responsible for loss in the value of compost as fertilizer [8,13]. Previous studies have shown a wide range of NH_3_ and N_2_O emissions, accounting for 9.6–46% and 0.1–5%, respectively, of the initial total nitrogen (TN) [14,15,16]. Sommer [17] and Parkinson et al. [18] reported that nitrogen losses in leachates ranged from 25–35% of initial TN while composting different types of manure.

N losses not only weaken the agronomic value of the composted product but also cause severe odor problems and increase greenhouse effects [19,20]; therefore, it is necessary to understand the pathways involved in N losses from various composting methods. Although several researchers have studied the effects of composting methods (mixed and unmixed compost) on nitrogen conservation and loss during composting of dairy manure and pig manure, these studies have focused on only one or two composting methods [21,22]. In terms of farmer compost (FC), which in this case is considered to be uncovered, the compost pile is subjected to ambient environmental conditions, which influence gaseous emissions and N leaching from the compost pile [23]. During pig manure composting, Szanto et al. [22] found that the method of mixed composting (turning the compost pile) increased NH_3_ volatilization and decreased N_2_O emissions compared with not turning the pile. However, in research on composting processes for cow manure, Ahn et al. [21] reported that the N_2_O production by a mixed compost (MC) method (turning the compost pile) was 3.5 times higher than that from an unturned pile. Sagoo et al. [24] reported that the anaerobic compost (AnC) method, which includes compacting and covering broiler litter heaps, decreased ammonia emissions by 90% and TN losses by 70% in comparison with conventional stockpiling. Shah et al. [25,26] found that N losses were reduced by approximately 72–78% by covering the heaps with an impermeable plastic sheet. N losses via leachates were less than 2% of the initial TN under relatively high aeration rates [8]. As composting technology has widely been adopted for the disposal of organic waste [10,14,27,28], it is essential to compare how these compost methods (e.g., FC, AnC, MC, and AC) influence nitrogen emissions and leaching losses during composting. To the best of our knowledge, no study has been conducted to investigate the effects of these four composting methods on nitrogen losses when composting dairy manure. Moreover, based on the benefits of covering compost in reducing nitrogen losses [24,25,26], only FC was not covered among the four composting treatments in our study.

Compost ventilation has a beneficial effect on nitrogen transformation and transfer [10,29], and aerobic composting is considered an effective way to manage livestock manure [30]; therefore, we hypothesized that AC would have positive effects on composting and decrease TN losses. The objectives of this study were to evaluate the potential effects of four composting methods on nitrogen retention and losses during composting and to find a suitable composting method that can both conserve nitrogen and prevent nitrogen losses.

## 2. Materials and Methods

### 2.1. Composting Experiments

This study was implemented for 39 days from 6 October to 13 November 2016. Dairy manure and rice straw were the raw materials, and they were collected from a dairy cow farm located in Dali, Yunnan, China, which covers an area of about one hectare. The rice straw was cut into 5–10 cm fragments. The moisture content and TN of the materials were measured before they were placed in the reactors. The properties of the materials were as follows: The TN levels of the dairy manure and rice straw were 21.2 and 6.1 g/kg, respectively, and the moisture content percentages of the dairy manure and rice straw were 80% and 5%, respectively.

There were four treatments in the study as follows: FC, AnC, MC, and AC (Table 1). Each treatment was conducted in four replicates that were carried out in four identical reactors. The initial amounts of the materials were as follows: 75 kg of fresh dairy manure and 15 kg of rice straw. The dairy manure and rice straw were mixed manually before they were loaded into the reactors. The experiment was carried out in composting reactors (Figures S1 and S2). Except for the AC treatment, the compost was loaded into the same reactors, which were 1.27 m^3^ iron cylinders (0.5 m high and 1.8 m inner diameter). Each reactor was divided into four vessels with the same volume. At the bottom of the reactors, there were four holes for leachate drainage. The reactor for the AC had two floors, and the sizes of the two reactors were the same as that for the other treatments. The bottom of the upper reactor had many equally-distributed holes to ensure uniform gas distribution. One side of the lower reactor was connected to a fan, and the bottom had holes for collecting leachate. One temperature sensor (RC-4 temperature recorder, Elitech, Xuzhou, China) was inserted into the middle of each compost load to record data at 9:00, 12:00, and 23:00 every day, and the values were then averaged. These hours were selected by referring to some other studies [31,32] and taking into account local actual experimental situation. The ambient temperature was also simultaneously recorded using the same temperature sensor and time of recording as the compost pile. In addition, the temperature sensor was placed near the compost reactor.

### 2.2. Compost Sampling and Chemical Analysis

Representative samples and leachates of the compost mixtures were collected on the following days: 1, 4, 8, 12, 16, 20, 24, 28, 32, and 39. The representative samples from each type of compost were divided into two parts; one part was stored at 4 °C until extraction to measure NH_4_^+^-N and NO_3_^−^-N, and the other part was air dried and ground to pass through a 1 mm sieve for further analysis. The dried and ground samples were subjected to analytical tests for TN. The leachates were all stored at 4 °C until extraction to measure TN. The moisture contents of the compost mixtures were determined by oven drying the samples at 105 °C until a constant weight was achieved. NH_4_^+^-N and NO_3_^−^-N in the compost were extracted with 2.0 M KCl (1:10 fresh solid sample to KCl, weight/volume) and determined using an automated colorimeter (SYSTEA S.p.A., Anagni, Italy). The TN in the leachates was quantified according to the standard method for water quality determination of Kjeldahl nitrogen (GB11891-1989).

### 2.3. N_2_O and NH_3_ Emission Measurements

Gas samples were taken from the surface of the compost piles on the following days: 1, 2, 4, 6, 8, 10, 12, 14, 16, 18, 20, 22, 24, 26, 28, 30, 32, 35, and 39. N_2_O was sampled using a static closed-chamber technique and was measured by a gas chromatograph that was equipped with both electron capture and flame ionization detectors [21]. The calculation for the N_2_O emission flux is below [33].

(1) $$F=\frac{\Delta m}{A\times \Delta t}=\frac{m2-m1}{A\times \Delta t}=\frac{C2\times V2\times M0\times \frac{273}{273+T2}-C1\times V1\times M0\times \frac{273}{273+T1}}{A\times \left(t2-t1\right)\times 22.4}$$

In the formula, *F* is the N_2_O emission flux, mg/(m^2^·h), and it is positive for emissions and negative for absorption. *A* is the area of the bottom of the sampling box, m^2^; *V* is the sampling box volume, m^3^; *m*_1_ and *m*_2_ are the masses of the gas in the box when the sample box is closed and opened, respectively; *t*_1_ and *t*_2_ are the times when the sampling box is closed and opened, respectively; *T*_1_ and *T*_2_ are the temperatures in the box when the sampling box is closed and opened, respectively; *C*_1_ and *C*_2_ are the volume fractions of greenhouse gases when the sample box is closed and opened, respectively; *M*_0_ is the molar mass of the gas, g/mol; and 22.4 is the gas molar volume at 101.325 kPa and 273 K, L/mol.

The NH_3_ emissions were successively captured by sponges immersed in 15 mL of a glycerophosphate solution [33,34]. Each sponge soaked in NH_3_ was immersed in 250 mL of a 1 mol/L KCl solution, and NH_3_ volatilization was determined after oscillation for 1 h. The calculation for NH_3_ volatilization is below [33].

(2) $$f=\frac{c\times v}{s\times t}$$

In the formula, *f* is the ammonia emission flux, mg/(m^2^·h); *C* is the content of ammonia nitrogen in the extract, mg/L; *V* is the volume of the extract, ml; *S* is the effective area of the sponge, m^2^; and *t* is the sampling time, h.

### 2.4. Germination Index (GI) Measurement

Aqueous compost extracts were obtained to determine the seed germination index (GI). Fresh solid samples were mixed with deionized water at a ratio of 1:10 (m/v) and shaken mechanically for 1 h [35]. The seed GI was used to assess phytotoxicity [36]; 20 rice seeds were uniformly distributed on filter paper in Petri dishes (10 cm diameter) and moistened with 8 mL of the compost extract. Four replicate dishes for each sample were incubated at 25 °C for 48 h. As a control, 8 mL of distilled water replaced the extract for every treatment. The number of germinating seeds and root length were measured. The GI was calculated by the following formula [37]:

(3) $$\mathrm{GI}\left(\%\right)=\frac{\mathrm{seeds}\;\mathrm{germination}\;\mathrm{of}\;\mathrm{treatment}\left(\%\right)\times \mathrm{root}\;\mathrm{length}\;\mathrm{of}\;\mathrm{treatment}}{\mathrm{seeds}\;\mathrm{germination}\;\mathrm{of}\;\mathrm{control}\left(\%\right)\times \mathrm{root}\;\mathrm{length}\;\mathrm{of}\;\mathrm{control}}$$

### 2.5. N Loss Calculation

The dry matter content was analyzed after drying the samples at 105 °C to a constant weight. TN was measured by the Kjeldahl digestion method [38]. Masses of N were determined (N concentration×weight of dry matter) [39], and N losses were computed as follows [40]:

(4) $$N\;\mathrm{losses}\left(\%\right)=\frac{N1\times M1-N2\times M2}{N1\times M1}\times 100$$

where *N*_1_ is the initial TN concentration, *N*_2_ is the final TN concentration, and *M*_1_ and *M*_2_ are the initial and final dry mass weights, respectively.

### 2.6. Statistical Analysis

All data were subjected to one-way analysis of variance (ANOVA), and multiple comparison tests were used to compare the least significance difference (LSD) at *p* = 0.05 using SPSS 22.0 software for Windows (IBM, Armonk, NY, USA). Sigma Plot software (Systat Software Inc., San José, CA, USA) was used for drawing.

## 3. Results

### 3.1. Compost Temperature and Moisture Content

**Figure 1:**
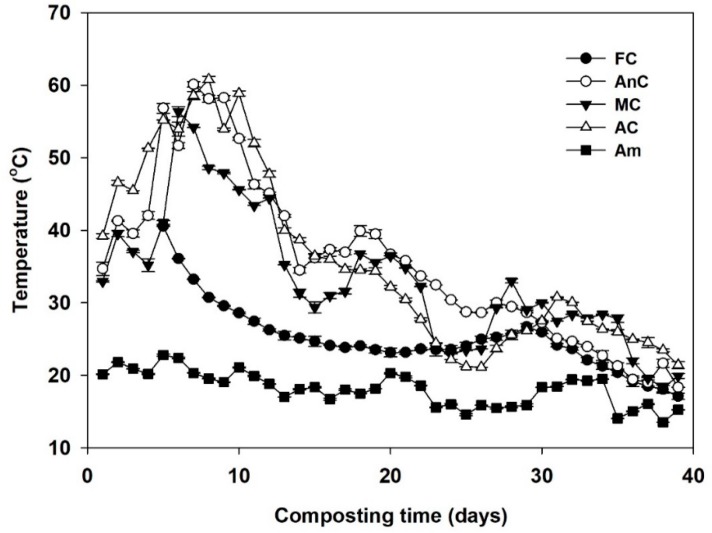
Changes in temperature of the composting material during dairy manure composting. FC—farmer compost; AnC—anaerobic compost; MC—mixed compost; AC—aerobic compost; Am—ambient levels. (The temperature values of the FC treatment in the first four days were abnormal due to instrumental failure and; therefore, are not shown).

The temperature throughout the composting period is presented in Figure 1. The ambient temperature ranged from 13–23 °C, and the studied mixtures except for FC demonstrated a typical temperature pattern for composting (heating, thermophilic, and cooling phases) and ranged from 17.1–60.8 °C. The high temperature periods (>50 °C) in the AnC, MC, and AC occurred on days 5, 6, and 4, and the duration of these periods lasted 6, 2, and 8 days, respectively. The maximum temperatures in the AnC, MC, and AC were 60.1, 56.4, and 60.8 °C and were observed on days 7, 6, and 8, respectively. After this elevation period, the temperature gradually decreased to ambient levels (Am), and this marked the end of the thermophilic phase of composting.

**Figure 2:**
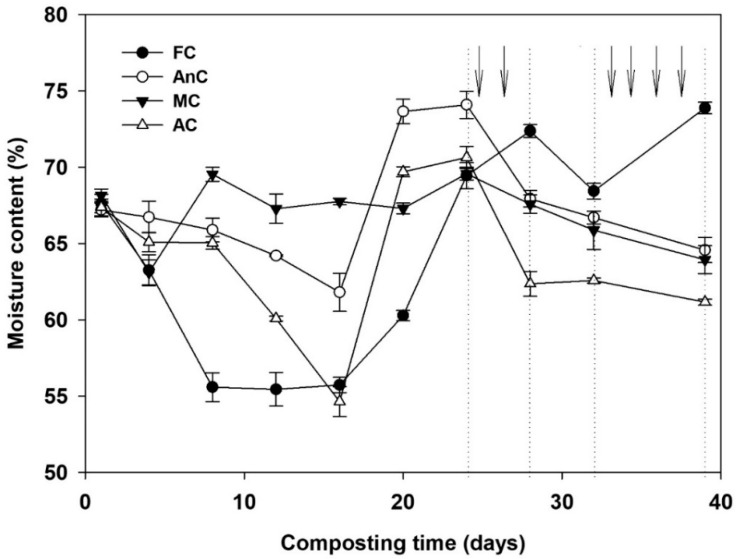
Changes in moisture content of the composting material during dairy manure composting. FC—farmer compost; AnC—anaerobic compost; MC—mixed compost; AC—aerobic compost. (The arrow indicates that there was rainfall).

The moisture levels throughout the composting period are presented in Figure 2. During the composting process, the moisture content of all treatments decreased rapidly, then increased, and finally showed a downward trend. The minimum moisture content was observed on days 12, 6, 2, and 16, and reached 55.4%, 61.8%, 63.1%, and 54.6%, for the FC, AnC, MC, and AC, respectively. At the end of the process, the moisture content was 73.9%, 64.6%, 64.0%, and 61.2% for the FC, AnC, MC, and AC, respectively, and the difference between the FC and the other compost treatments was significant (*p* < 0.05).

### 3.2. Germination Index

**Figure 3:**
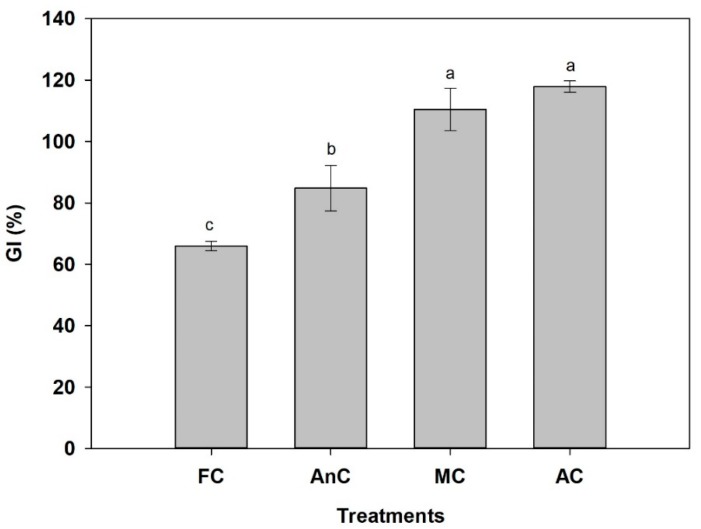
Germination indexes (GIs) of different composting treatments at the end of the process. FC—farmer compost; AnC—anaerobic compost; MC—mixed compost; AC—aerobic compost. Data are shown as the mean ± SD. Bars with different letters mean significant difference at *p* < 0.05.

The GIs of the compost products are presented in Figure 3. At the end of the process, the GIs of the AnC, MC, and AC were 84.8%, 110.4%, and 117.9%, respectively. The GI of the FC was the lowest at 65.9%, which was less than 80%.

### 3.3. Nitrogen Gas Emissions

**Figure 4:**
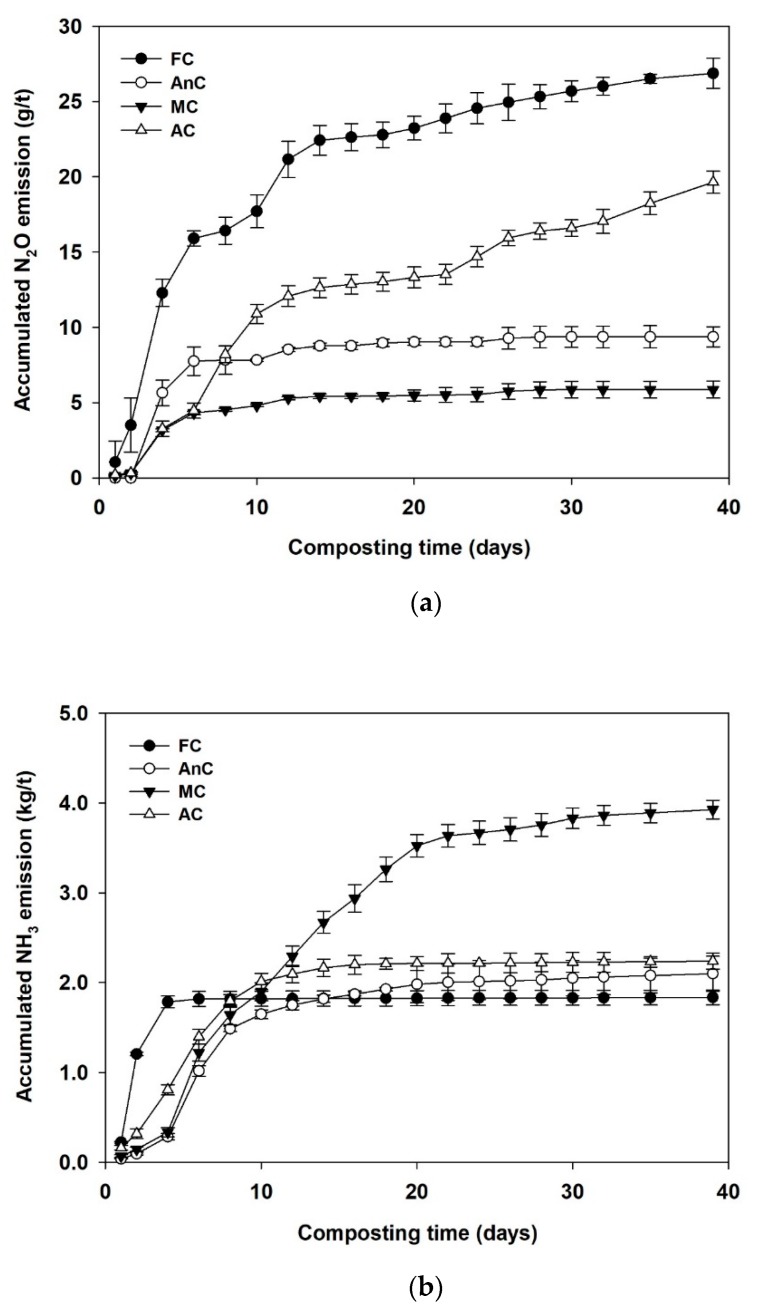
Accumulated gaseous emission of N_2_O (**a**) and NH_3_ (**b**) during dairy manure composting. FC—farmer compost; AnC—anaerobic compost; MC—mixed compost; AC—aerobic compost.

As shown in Figure 4a, the N_2_O emissions were mostly concentrated in the first two weeks of the study for all the treatments. In the later stage of composting, the N_2_O emissions of the AnC still increased slowly. During the whole composting process, the N_2_O emission rate of the MC was the lowest, with total emissions of 5.86 g/t. Higher cumulative N_2_O emissions were recorded for the FC (26.87 g/t) in comparison with that in the AC (19.64 g/t) and AnC (9.37 g/t).

During the composting process, the amount of gaseous NH_3_ emissions was similar to that of the N_2_O emissions (Figure 4b). The accumulated NH_3_ emissions from the MC continued to increase throughout the composting process, while the accumulated NH_3_ emissions from the FC, AnC, and AC stabilized on days 6, 22, and 16 of composting, respectively. At the end of composting, the total amounts of NH_3_ emission from the FC, AnC, MC, and AC were 1.84, 2.10, 3.93, and 2.24 kg, respectively.

### 3.4. Nitrogen Variations in the Composts

**Figure 5:**
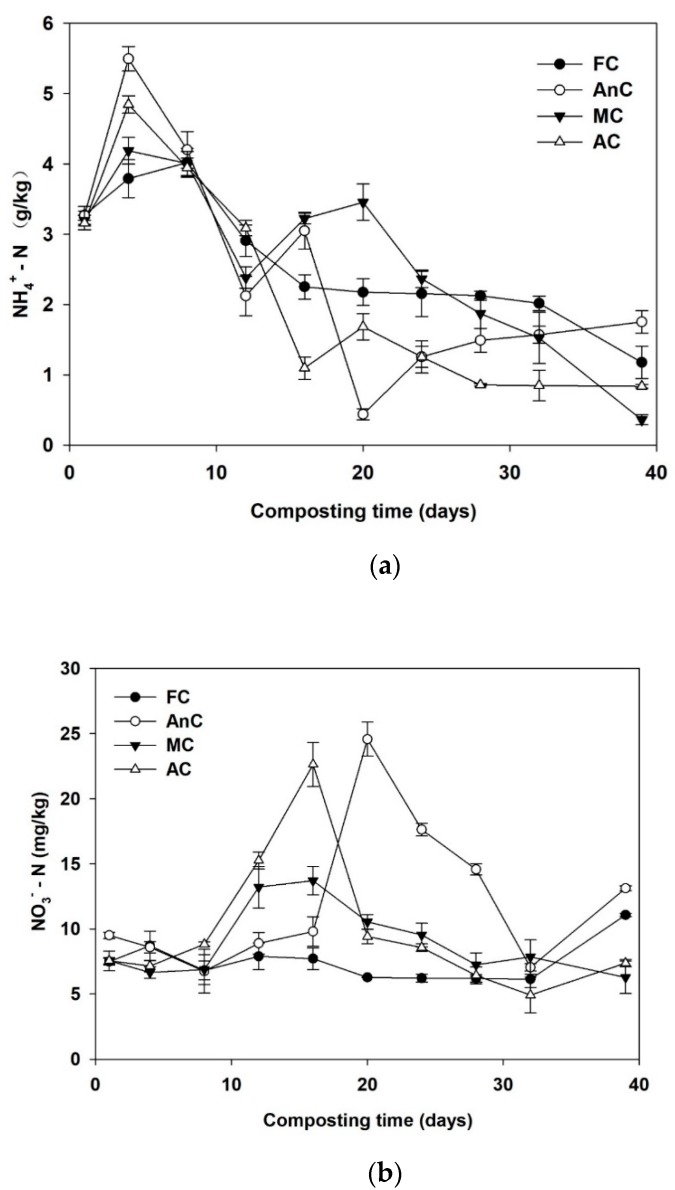

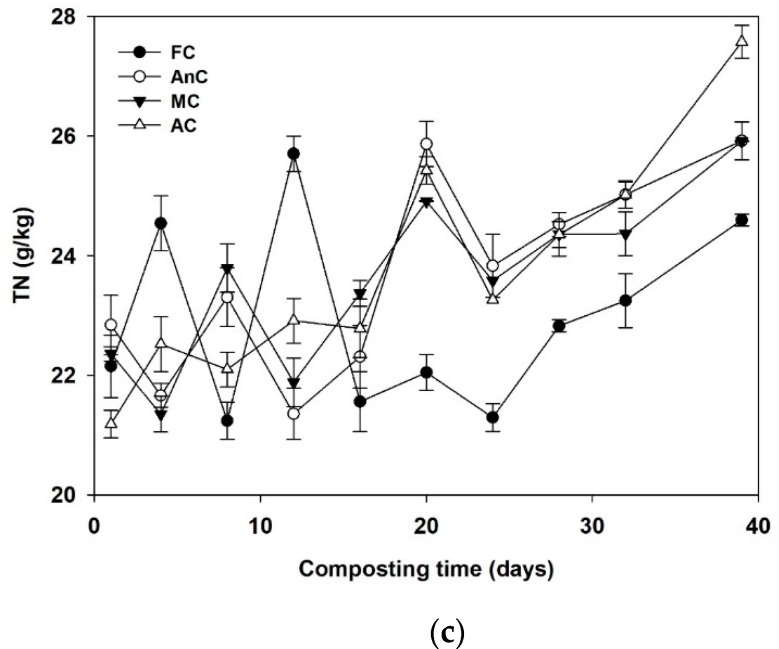
Changes in NH_4_^+^-N (**a**), NO_3_^−^-N (**b**), and TN (**c**) concentrations in the compost material during dairy manure composting. FC—farmer compost; AnC—anaerobic compost; MC—mixed compost; AC—aerobic compost.

As shown in Figure 5a, the initial NH_4_^+^-N concentrations of the treatments were approximately 3.22 mg/g. The NH_4_^+^-N concentrations of all the treatments (FC, AnC, MC, and AC) increased during the first week and peaked at days 7, 4, 4, and 4, respectively. In addition, the NH_4_^+^-N concentrations of all the treatments (FC, AnC, MC, and AC) increased by 22.9%, 68.2%, 31.0%, and 53.3%, respectively, in comparison to their initial values. Subsequently, the NH_4_^+^-N concentrations gradually decreased in all the treatments until the end of composting. However, higher NH_4_^+^-N contents were observed in the FC and AnC than in the MC and AC.

The initial NO_3_^−^-N concentrations were very low for all the treatments (8.02 mg/kg) (Figure 5b). The NO_3_^−^-N concentrations did not change significantly until day 7 (2.07–2.50 mg/kg). After that day, the NO_3_^−^-N concentrations in all the treatments, except that in the FC, rapidly increased. However, the NO_3_^−^-N concentrations in the FC only slightly changed. At the end of composting, the increases in TN concentrations in the FC, AnC, MC, and AC were 11.0%, 13.5%, 15.9%, and 30.2%, respectively.

During the initial stage of the composting process, the TN content in all the treatments fluctuated slightly and then gradually increased after the thermophilic phase (Figure 5c). The TN content in the AC (27.57 g/kg) was significantly (*p* < 0.05) higher than that in the other treatments (FC 24.60 g/kg, AnC 25.92 g/kg, and MC 25.92 g/kg) at the end of composting. However, the TN content in the FC was significantly lower than those in the other treatments.

### 3.5. Volume and TN Variations in the Leachate

**Figure 6:**
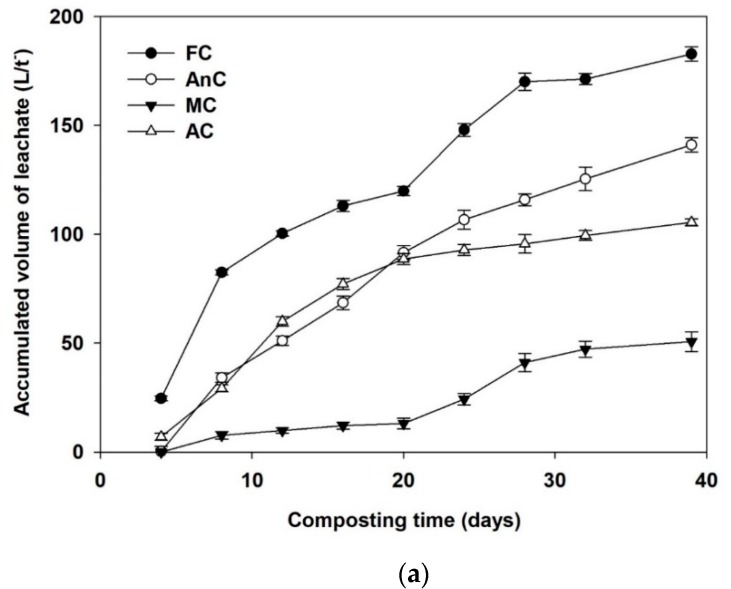

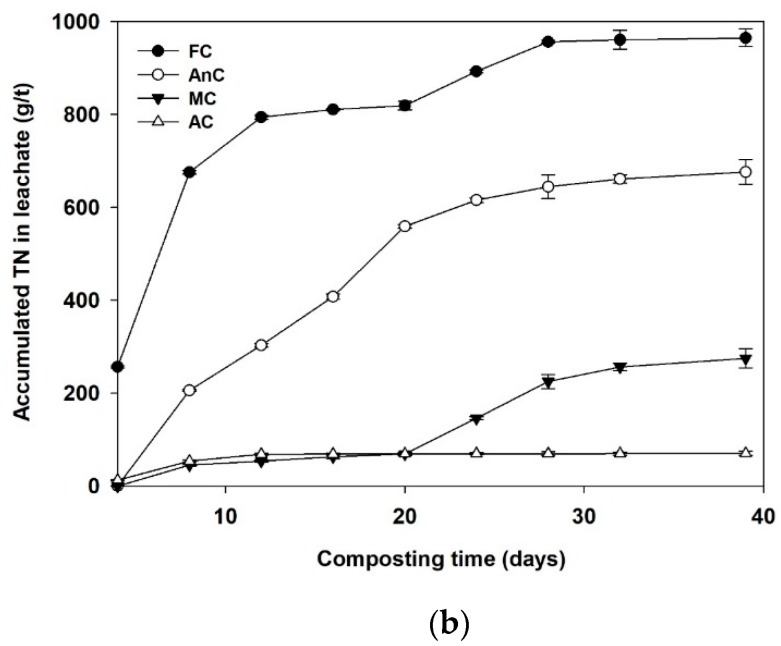
Accumulated volume (**a**) and total nitrogen (TN) (**b**) in the leachate during dairy manure composting. FC—farmer compost; AnC—anaerobic compost; MC—mixed compost; AC—aerobic compost.

The accumulated leachate volumes during the composting are presented in Figure 6a. During the whole composting process, the amount of leachate from the FC was greater than that from the other treatments. The amount of leachate from the FC and AnC continued to increase throughout the composting process, while the accumulated volumes of leachate from the MC and AnC stabilized after 28 days of composting. At the end of composting, the cumulative amounts of leachate in the FC, AnC, MC, and AC were 182.8, 141.0, 50.67, and 105.46 L/t, respectively.

During the whole composting process, the TN loss in the leachate from the AC was always the lowest, and TN mostly no longer increased beginning on the twelfth day of composting (Figure 6b). At the beginning of composting, the TN losses in the leachate from the FC and AnC significantly increased, while the TN loss was relatively slow in the later stages of composting. The TN loss in the leachate from the FC and AnC was very stable after 28, 30, and 32 days. At the end of composting, the TN losses in the leachate were 964.7, 676.1, 274.8, and 70.4 g/t for FC, AnC, MC, and AC, respectively.

### 3.6. TN Losses

The N losses from all the treatments are presented in Table 2. The N losses during composting mainly occurred via three mechanisms: NH_3_ volatilization under high temperatures and high pH values, NO_x_ volatilization caused by nitrification and denitrification, and water-soluble nitrogen loss via the leachate. At the end of composting, the cumulative N losses via N_2_O emissions, NH_3_ emissions, leachates, and other means accounted for 0.02–0.09%, 8.10–17.33%, 0.38–5.17%, and 0.91–2.81%, respectively, of the initial TN. The N losses from the MC were the highest, up to 19.75%, in comparison with the N losses from the other composting methods. The N losses from the FC, AnC, and AC were 15.98%, 15.08%, and 13.13%, respectively.

## 4. Discussion

### 4.1. Effects of Different Composting Treatments on Compost Temperature, Moisture Content, and GI

Temperature was used as one of the main parameters to monitor the performance of the composting process [41,42]. Previous research demonstrated that the heat from composting was generated via the rapid consumption of easily-degradable organic material, which was used as nutrients for microbial activity and growth [43]. In the first week of composting, the microorganisms rapidly decompose the degradable organic matter in the material and release energy as heat, causing the compost temperature to sharply rise [44]. After the thermophilic phase, the decomposition rate decreased as the organic matter in the pile became more stabilized, with a consequent decrease in temperature and microbial activities [41,45]. The temperatures of the AnC and AC remained above 50 °C for more than five days, reaching the high-temperature composting requirements (GB7959-1987). The FC and MC did not meet the criteria, considering that the thermophilic phase was characterized by a high rate of biodegradation [42,46], which may have been because the degradation rates of their organic matter were low in the thermophilic phase.

Moisture was kept at the appropriate level to allow proper composting performance, as moisture has a significant effect on microbial activity and temperature [41]. Although the optimum initial moisture content for composting is generally between 55–65% [47], Li et al. [48] found that the higher initial moisture content (65%) was more favorable for its higher temperature, longer retention time of high temperature, and the more stable end compost obtained. In addition, many researchers [40,49] also chose the higher initial moisture content (>65%) during the composting process. Some studies have reported that their moisture content was approximately 70% when composting and that microbial activity was the highest at this moisture content [50]. In this experiment, the moisture content of all the treatments reached approximately 60–70%, which provided a suitable aqueous environment for the microorganisms. The high temperature during composting was an important cause of the sharp decline in the compost moisture content. The moisture content of the FC treatment was affected by evaporation and rainfall due its uncovered state. As indicated by the arrow in Figure 2, during the first 24–28 and 32–39 days of composting, while the moisture content of the other three treatments decreased, the FC moisture content continued to increase. During the first 20–24 days of composting, water was added to the AnC, MC, and AC to increase the moisture content of these materials. At the end of composting, the AC moisture content was the lowest among all the treatments, which may be due to the longer duration of the high-temperature period for this composting method than for other composting methods. Additionally, the FC moisture content was significantly higher than that of the other three treatments (*p* < 0.05), which was mainly caused by rainfall.

The GI was an important biological indicator for evaluating compost maturity and phytotoxicity [37]. A GI of more than 80% indicates nonphytotoxic and mature compost [51]. The GIs of the AnC, MC, and AC treatments were more than 80%, but the GI of the FC was only 65.9%. The GI is known to increase with the decomposition of toxic material [52]. The low GI of the FC could be caused by low temperature (Figure 1), which restrained the microorganism activity [35]; as a result, the decomposition of toxic materials, such as short chain volatile fatty acids (mainly acetic acid) in the compost was lower than in the other three treatments [40,52]. In addition, the high electrical conductivity (EC) values might be the reason for the relatively low GI [35].

### 4.2. Effects of Different Composting Treatments on Nitrogen Retention

The changes in NH_4_^+^-N reflected the nitrogen transformations and NH_3_ emissions [40]. The NH_4_^+^-N concentrations of each treatment sharply increased and then gradually decreased and stabilized during the composting process. The increases in NH_4_^+^-N concentrations in all treatments during the first several days of the process were related to the rapid mineralization of organic matter and ammonification during the bio-oxidative phase of composting [53]. The decrease in NH_4_^+^-N concentrations resulted from the conversion of NH_4_^+^-N to volatile NH_3_, the immobilization of nitrogenous compounds such as amino acids, nucleic acids, and proteins by microbes [54], and the conversion of NH_4_^+^-N to NO_3_^−^-N [55]. Because it was uncovered, the FC was subjected to ambient environmental conditions (i.e., rainfall, temperature, wind, and radiation). The low NH_4_^+^-N of the FC in the thermophilic phase could be caused by low temperature (Figure 1), which restrained the microorganism activity [35], as a result; the degradation rates of its organic matter were lower than those of the other three treatments in the thermophilic phase [42,46]. Compared with the AnC, the MC and AC were turned during the composting process, which promoted the volatilization of NH_3_ (Figure 4b), resulting in decreased NH_4_^+^-N concentrations (Figure 5a). In addition, the higher NH_4_^+^-N concentrations in the FC and AnC than in the MC and AC might be due to the relatively high moisture contents of the FC and AnC (Figure 2), which allowed them to absorb some NH_3_, thereby impeding NH_3_ volatilization [56].

Because the growth and activity of nitrifying bacteria and nitrobacteria were inhibited from excessive NH_4_^+^-N accumulation and high temperatures [57], the NO_3_^−^-N concentration did not significantly change until day 7. With the decrease in compost temperature, the volatilization of a large amount of NH_3_ from the compost and the enhanced nitrifying bacterial metabolism, NH_4_^+^-N was gradually transformed into NO_3_^−^-N [1]. At the end of composting, the NO_3_^−^-N concentrations in the FC and AnC were increased by 38.8% and 63.8%, respectively, in comparison with the NO_3_^−^-N concentrations before composting, and the NO_3_^−^-N concentrations in the MC and AC were reduced by 21.3% and 7.5%, respectively. The NO_3_^−^-N concentration of each treatment was relatively low during the whole composting process. Differences in the NO_3_^−^-N concentrations have been reported by many researchers [1,58,59]. Jiang et al. [1] and Ren et al. [40] found that NO_3_^−^-N was very low (<0.5 g/kg) during the whole composting process. Li et al. [49] reported that the highest NO_3_^−^-N levels reaching 1.09 g/kg in all studied treatments. In our study, the NO_3_^−^-N concentration was between 5 to 25 mg/kg during the whole composting process. Similarly, Sanchez-Garcia et al. [58] obtained the same results as we did, and the NO_3_^−^-N concentration was lower than 50 mg/kg during composting of poultry manure. Additionally, He et al. [60] and Li et al. [59] found that the NO_3_^−^-N concentrations in all their studied treatments were lower than 100 and 120 mg/kg, respectively.

The changes in TN content in the compost material were affected by a variety of factors. For instance, NH_3_, which is produced by the decomposition of organic nitrogen, is volatile at high temperatures, resulting in a decrease in TN contents. The enrichment effect of the strong degradation of organic compounds increased the TN content of the compost [1,53], which reduced the total dry mass and concentrated the nitrogen [49]. At the end of composting, in comparison with the TN contents from the other composting methods, the TN content of the AC was the highest, indicating that AC treatment can effectively reduce nitrogen losses.

### 4.3. Effects of Different Composting Treatments on Nitrogen Losses

N_2_O emissions are mainly associated with nitrification and denitrification processes [61]. Compared with the N_2_O emission rate at the end of composting, in the early stage of composting, the N_2_O emission rate was higher for all the treatments, except that for the MC, mainly due to the higher initial temperature during composting coupled with the NO_3_^−^ and NO_2_^−^ in the compost materials. Under these environmental conditions, microbial denitrification was abundant, and N_2_O was continuously discharged. In addition, the nitrification of ammonium nitrogen in high-temperature conditions can also produce N_2_O via methane-oxidizing bacteria [22]. Chowdhury et al. [29] suggested that biological nitrification is probably a major contributor to the increased N_2_O emission rates at the beginning of the thermophilic phase, meaning that both nitrification and denitrification processes coexist in compost piles. In the high-temperature phase, AnC had a higher N_2_O emission rate than AC due to the anaerobic environment. In contrast, in the cooling phase, higher cumulative N_2_O emissions were recorded for the AC than in the AnC (Figure 4a), which may be because an aerobic environment was provided in the AC. At the end of composting, there were significant differences among the four composting treatments in terms of cumulative N_2_O losses (*p* < 0.05). The cumulative N_2_O losses from the MC were the lowest at only 5.86 g/t. Because it was uncovered, the FC was subjected to ambient environmental conditions (i.e., rainfall, temperature, wind, and radiation). Additionally, the FC had significantly higher cumulative N_2_O losses (by 36.84% and 186.93%, respectively) than the AC and AnC, which might be because a more suitable environment for nitrification and denitrification was created in the FC than in the AC and AnC.

The NH_3_ emissions of the four treatments were mainly concentrated during early composting, which was consistent with the results from previous studies [62]. This result was mainly due to rapid microbial metabolism during the early stage of composting, and the decomposition of organic nitrogen compounds produced a large amount of ammonium nitrogen, thus accelerating the NH_3_ emissions from the composting process [63]. Subsequently, the compost temperature gradually decreased, the microbial activity declined, and the NH_3_ emissions also decreased. High temperatures and pH values promote NH_3_ volatilization during the thermophilic phase [53]. However, unfavorable conditions, such as high moisture conditions, prevent the conversion of NH_4_^+^ to NH_3_ [56]. In this environment, the cumulative NH_3_ emissions in the FC, AC, and AnC were significantly lower than those in the MC (*p* < 0.05).

Yang et al. [9] reported that because of a high water content and compact structure in the compost pile, wastewater readily leached out of their compost and formed leachates. In addition, the types of composting material, the amount of water they can hold, and the ventilation conditions can greatly influence leachate release [8]. At the end of composting, the cumulative leachate volumes from the FC (182.75 L/t) and AnC (141.02 L/t) were significantly higher than those from the AC (105.46 L/t) and MC (50.67 L/t) (*p* < 0.05). This result was probably because the FC was not covered by plastic film during the composting process, and rainfall led to the highest amount of leachate. The leachate discharge from the FC treatment was mainly concentrated in the early stage of composting, which was also the main reason for the lower composting temperature in the FC treatment than in the other treatments.

Leachate quality has rarely been studied. Relevant studies have indicated that compost leachate is variable in terms of its chemical composition, which is influenced by factors such as the nature of the feedstock, the composting technology employed, the degree of cover, and the weather [64]. The TN content of compost leachate usually varies with the characteristics of the composting materials, compost maturity, and composting process [65]. At the end of composting, the TN losses in leachate from the FC (964.7 g/t) and AnC (676.1 g/t) were significantly higher than those from the MC (274.8 g/t) and AC (70.4 g/t) (*p* < 0.05). The loss of TN in leachates was mainly related to the accumulated discharge of leachates [8]. In addition, the loss of TN in leachates was also related to the loss of nitrogen in the form of gas (NH_3_ and N_2_O) from the compost.

### 4.4. Effects of Different Composting Treatments on N Balances

Many previous studies have examined N losses during composting and have evaluated these losses under different composting conditions [29,66,67], and they suggested that high N losses could be explained by high temperatures and the long duration of the thermophilic phase. In this study, the accumulated N_2_O emissions from N losses were rather low. The cumulative N losses via N_2_O emissions accounted for only 0.02–0.09% of the initial TN, and this result was within the range of values (<1% of the initial TN) reported in a number of published studies summarized by Brown et al. [68] and was also in agreement with the results from several additional previous studies [10,29,69,70]. NH_3_ volatilization accounted for 8.10–17.33% of the initial TN, which was comparable to the values reported in previous studies [29]. The cumulative N losses via leachates accounted for 0.38–5.17% of the initial TN. Other forms of nitrogen losses during the composting process were mainly through N_2_, and the range of other forms of nitrogen losses in our study was consistent with the results from Zeng et al. [10]. At the end of composting, the AnC reduced N_2_O and NH_3_ volatilization to a certain extent, but the cumulative N losses via leachates were higher in the AnC than in the other treatments. N_2_O volatilization and leachate discharge were both the lowest in the MC compared with the other composting methods, but the cumulative N losses via NH_3_ volatilization were the highest in the MC. Our results showed that the AC had the lowest nitrogen losses and that the differences between the AC and FC as well as MC were significant (*p* < 0.05). However, Shah et al. [26,71] concluded that AnC treatment resulted in the lowest amount of N losses in their study. The main cause of this difference was the different composting conditions. In their study, the AnC was covered with a plastic sheet, and aerobic composting was accomplished through frequently mixing the compost in open air. However, in our study, the aerobic compost was frequently turned and covered with plastic film, and fresh air was pumped into the compost by a fan for 30 min daily. Considering these findings, turning, covering, and ventilating compost can significantly reduce N losses via leachates, further reducing TN losses, and this result also supports our proposed hypothesis.

## 5. Conclusions

Among the four compost treatments, the FC had not only the highest N losses but also the lowest GI (only 65.9%). However, the GIs of the other three compost treatments exceeded 80%, meeting the maturity requirements for composted products. The AnC treatment reduced N_2_O and NH_3_ volatilization to a certain extent, but the cumulative N losses via leachates were higher in this treatment than in the other treatments. N_2_O volatilization and leachate discharge were both lower in the MC than in the other composting methods, but the cumulative N losses via NH_3_ volatilization were the highest in the MC. In addition, under the studied conditions, the FC and MC did not reach a temperature that would allow the compost to be disinfected. The N_2_O and NH_3_ emissions from the AC were not the lowest among the studied composting methods; however, the N losses via leachates were significantly lower, and the TN loss rate was the lowest in the AC compared with the other composting methods.

Based on the results of this study, the AC method could be recommended due to its ability to significantly reduce N losses via leachates, which would clearly reduce soil, surface water, and groundwater pollution, thereby offering a potential strategy to reduce nitrogen losses, improve the quality of compost, and provide environmental benefits.

## Figures and Tables

**Table 1:** Experimental design.

Treatment	Specific Measures
FC	The famer’s method of composting was adopted, and the compost pile was not turned, ventilated, or covered with plastic film.
AnC	During the whole composting process, the compost pile was not turned or ventilated but was covered with plastic film.
MC	During the composting process, the compost pile was turned manually every 5 days and covered with plastic film but was not ventilated.
AC	Fans (120 W) actively pumped fresh air through perforated tubes at the bottoms of the reactors for 30 min daily, and the applied aeration rate was 2.5 m^3^/min. Moreover, the compost pile was thoroughly manually turned every 5 days and covered with plastic film.

FC—farmer compost; AnC—anaerobic compost; MC—mixed compost; AC—aerobic compost.

**Table 2:** N mass balance for the compost mixtures in each treatment during the 39-day composting period.

Treatment	N Losses (%)				
	N Losses Via N_2_O	N Losses Via NH_3_	N Losses Via Leachate	N Unaccounted for ^1^	TN Losses
FC	0.09 ± 0.008a	8.10 ± 1.35b	5.17 ± 1.17a	2.62 ± 0.43a	15.98 ± 1.99b
AnC	0.03 ± 0.009c	9.27 ± 0.98b	3.62 ± 0.38b	2.15 ± 0.75a	15.08 ± 1.66bc
MC	0.02 ± 0.007c	17.33 ± 0.61a	1.49 ± 0.10c	0.91 ± 0.26b	19.75 ± 0.57a
AC	0.07 ± 0.013b	9.88 ± 0.55b	0.38 ± 0.08c	2.81 ± 0.83a	13.13 ± 1.43c

FC—farmer compost; AnC—anaerobic compost; MC—mixed compost; AC—aerobic compost. Data represent means ± SD. Different letters indicate significant differences between the treatments; ^1^ N unaccounted for = TN losses − N_2_O Losses − NH_3_ losses − leachate losses.

## Supplementary Materials

The following are available online at https://www.mdpi.com/1660-4601/16/18/3324/s1, Figure S1: Schematic diagram of composting reactor, Figure S2: Photo of composting reactors.
