# Supplementary material for: Impact of Composting Methods on Nitrogen Retention and Losses during Dairy Manure Composting
Source: Int J Environ Res Public Health. 2019 Sep 9;16(18):3324. doi: 10.3390/ijerph16183324 (PMC6765887; doi:10.3390/ijerph16183324)
Supplement: Supplementary file 1 [file ijerph-16-03324-s001.pdf]

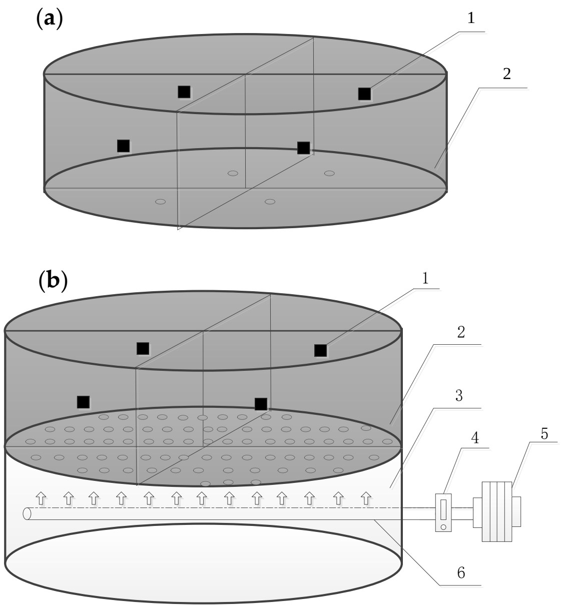

Figure S1. (**a**) Schematic diagram of composting reactors for farmer compost, anaerobic compost and mixed compost**;** (**b**) Schematic diagram of composting reactors for aerobic compost; 1—temperature sensor; 2—Pile layer; 3—Air distribution layer; 4—Rotameter; 5—Air pump; 6—Air distributor.

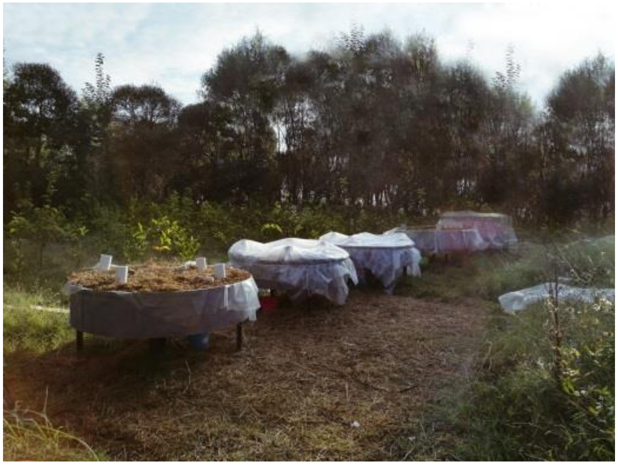

Figure S2. Photo of composting reactors.
